# *EGFR *G719A突变合并*LMNA*-*NTRK1*融合变异的肺腺癌1例病例报道

**DOI:** 10.3779/j.issn.1009-3419.2025.106.03

**Published:** 2025-01-20

**Authors:** Shiqi SONG, Yaxian YANG, Weiquan LUO, Yueya LIANG, Qi LI, Tongxu ZHUO, Weibin XIONG, Jian HUANG

**Affiliations:** ^1^524001 湛江，广东医科大学附属医院病理诊断与研究中心; ^1^Department of Pathological Diagnosis and Research Center, the Affiliated Hospital of Guangdong Medical University, Zhanjiang 524001, China; ^2^510730 广州，广州华银康医疗集团股份有限公司; ^2^Guangzhou Huayin Health Medical Group Co., Ltd, Guangzhou 510730, China; ^3^524000 湛江，湛江市妇幼保健院病理科; ^3^Department of Pathology, Maternal and Child Health Hospital of Zhanjiang City, Zhanjiang 524000, China

**Keywords:** 肺肿瘤, NSCLC, *EGFR*突变, *NTRK*融合, Lung neoplasms, NSCLC, *EGFR* mutation, *NTRK* fusion

## Abstract

神经营养受体酪氨酸激酶（neurotrophic receptor tyrosine kinase, *NTRK*）的融合变异是多种成人和儿科恶性实体瘤的致癌驱动因素，如乳腺癌、唾液腺癌以及婴儿纤维肉瘤等。*NTRK1/2/3*的基因重排导致原肌球蛋白受体激酶（tropomyosin receptor kinase, TRK）结构域的组成型激活，表达的融合蛋白驱动肿瘤生长和存活。在非小细胞肺癌（non-small cell lung cancer, NSCLC）中，*NTRK*融合发生的频率为0.1%-1%。表皮生长因子受体（epidermal growth factor receptor, *EGFR*）在NSCLC中为常见突变，但*EGFR* G719A突变发生频率较低（约2%），且*EGFR*突变通常和*NTRK*融合变异互斥。本文首次报道1例原发性肺腺癌组织中同时伴有*EGFR *G719A突变和*LMNA-NTRK1*融合变异的罕见病例，并通过文献复习探讨*NTRK*融合变异在NSCLC中的作用以及与*EGFR*突变之间的关系，以提高对*NTRK*融合变异NSCLC的认识。

## Abstract (en)

Fusion variations of neurotrophic receptor tyrosine kinase (*NTRK*) are oncogenic drivers in various solid tumors such as breast cancer, salivary gland carcinoma, infant fibrosarcoma, *etc*. Gene rearrangements involving *NTRK1/2/3* lead to constitutive activation of the tropomyosin receptor kinase (TRK) domain, and the expressed fusion proteins drive tumor growth and survival. *NTRK* fusions are estimated to occur at a frequency of approximately 0.1% to 1% in non-small cell lung cancer (NSCLC). Epidermal growth factor receptor (*EGFR*) mutations are prevalent in NSCLC, but the frequency of *EGFR* G719A mutation is relatively low (about 2%), and *EGFR* mutations are typically mutually exclusive with *NTRK *fusion variants. The study presented the first documented case of lung adenocarcinoma harboring both *EGFR* G719A mutation and *LMNA-NTRK1 *fusion. A review of the literature was conducted to elucidate the role of *NTRK *fusion mutations in NSCLC and their relationship with *EGFR *mutations, aiming to enhance the understanding of *NTRK* fusion mutations in NSCLC.

肺癌是全球癌症死亡相关的最主要原因。近年来，精准医疗进入肿瘤诊断和治疗领域，尤其是肺癌分子谱系分析及多个以肿瘤驱动突变为靶点的靶向治疗，为肺癌的治疗提供了更为有效的方法^[1]^。在非小细胞肺癌（non-small cell lung cancer, NSCLC）中神经营养受体酪氨酸激酶（neurotrophic receptor tyrosine kinase, *NTRK*）基因家族为少见突变，*NTRK*家族包括*NTRK1*、*NTRK2*和*NTRK3*基因，分别编码原肌球蛋白受体激酶（tropomyosin receptor kinase, TRK）家族蛋白质TRKA、TRKB和TRKC^[2]^。近年来学者^[3]^普遍认为，*NTRK*基因融合是多种肿瘤的致癌驱动因素，但在NSCLC中较为罕见，远低于*NTRK*基因在其他类型肿瘤中的突变频率。*NTRK*突变在NSCLC的常见融合伴侣包括*ETV6-NTRK3*、*TPM3-NTRK1*和*SQSTM1*。Wang等^[4]^报道了1例NSCLC经奥希替尼（Osimertinib）（靶向*EGFR *19del和T790M突变）靶向治疗后耐药并发现获得性*LMNA-NTRK1*融合的病例，*LMNA-NTRK1*原发性融合变异在NSCLC中尚未见报道。在NSCLC中表皮生长因子受体（epidermal growth factor receptor, *EGFR*）的常见突变类型主要为19del和L858R突变，其他少见突变包括G719X、外显子18插入、外显子19插入、外显子20 S786I、外显子20插入、外显子21 L861Q，以及外显子18-25的激酶结构域发生重复和重排（*EGFR-RAD51*和*EFFR-PURB*）等，大多数临床试验没有包括这些亚群^[5]^。研究^[6]^表明，*NTRK1*融合可能是EGFR-酪氨酸激酶抑制剂（tyrosine kinase inhibitors, TKIs）的耐药机制之一。原发性*EGFR* G719A突变合并*NTRK1*融合的肺腺癌尚未见报道。本文报道1例*EGFR* G719A突变合并*LMNA-NTRK1*融合变异的肺腺癌患者临床资料，旨在为特殊变异的肺腺癌患者的诊断和治疗提供借鉴。

## 1 病例资料

患者女性，59岁。2023年6月患者因应用“氨基比林”出现过敏反应入院。胸部增强计算机断层扫描（computed tomography, CT）显示右肺下叶后基底段肿块状软组织密度影（51 mm×34 mm），考虑为肺癌，周围见肺动脉小分支包饶，邻近右下肺静脉包饶受压；双侧锁骨上窝、纵隔内（2R、4R、5区、7区）、双肺门见多发增大淋巴结影，较大者位于4R区，短径约10 mm，右肺上叶结节轻度强化（图1）。此外，头颅磁共振成像（magnetic resonance imaging, MRI）显示右侧额颞部异常信号，结合病史考虑转移性病变。患者在CT引导下经皮肺穿刺活检术进行组织病理检查，镜下显示典型的乳头状（红色框）和微乳头状结构（绿色框）的癌巢呈浸润性生长[苏木精-伊红（hematoxylin and eosin, HE）染色切片形态特点见图2]，为肺腺癌，临床分期为cT4N3M1b IVA期。

**图1:**
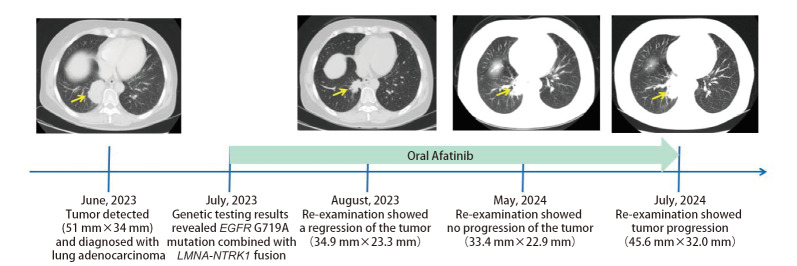
原发性肺腺癌进程CT影像学表现

**图2:**
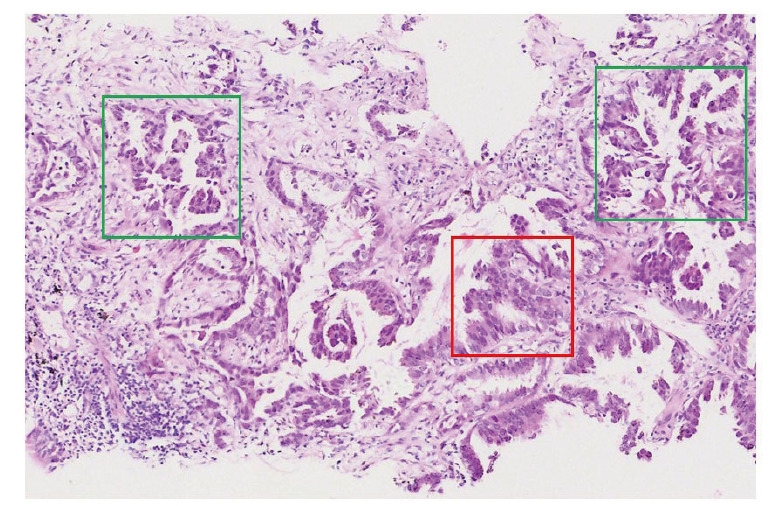
HE病理切片显示为肺腺癌（×100）。红色框为乳头状癌区域，绿色框为微乳头状癌区域。

2023年7月对肺腺癌组织切片进行DNA/RNA-based下一代测序（next generation sequencing, NGS）基因检测，结果显示：*EGFR* G719A变异（exon 18 c.2156G>C，突变丰度33.92%）；纤层蛋白A/C（lamin A/C, LMNA）（exon 2）与*NTRK1*（exon 11）发生融合变异（图3A），即*LMNA-NTRK1*融合变异[*LMNA *(chr1:156100564)-*NTRK1 *(chr1:156844698) L2N11]，融合方式见图3B。另外，补充了荧光原位杂交（fluorescence *in situ* hybridization, FISH）的验证，如图3C所示NTRK断裂探针显示分离信号，表示*NTRK*融合阳性。

**图3:**
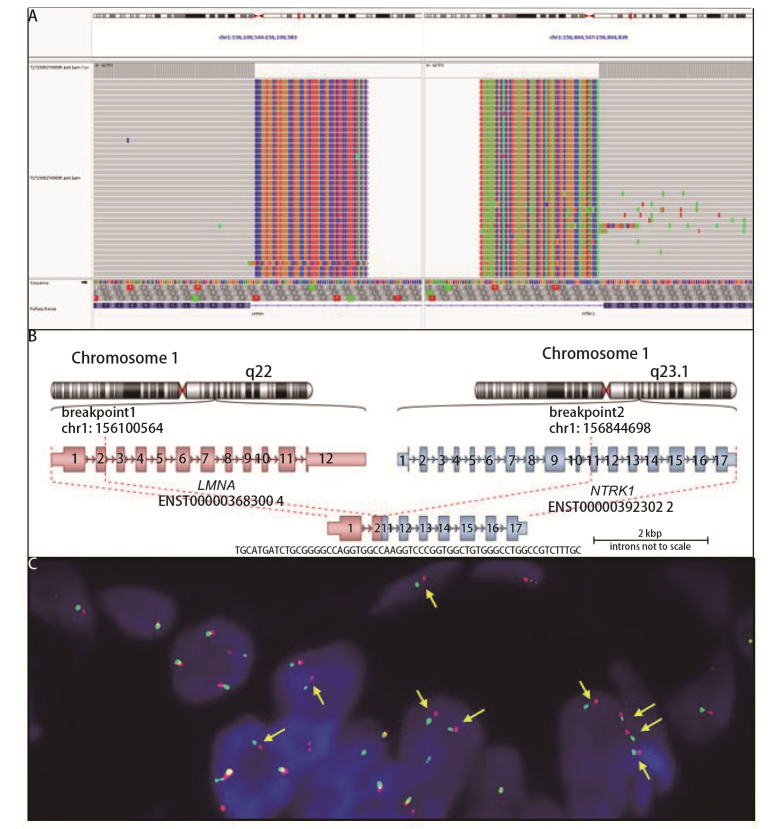
LMNA-NTRK1融合示意图。A：LMNA（ENST00000368300.4）-NTRK1（ENST00000392302.2）L2N11融合IGV截图；B：LMNA-NTRK1融合模式示意图；C：LMNA-NTRK1融合FISH验证（黄色箭头指示NTRK基因断裂）。

由于NGS检测结果发现*EGFR* G719A突变，患者于2023年7月开始口服阿法替尼（Afatinib）靶向治疗（30 mg *qd*）。1个月后复查CT显示右肺下叶病变缩小（34.9 mm×23.3 mm），治疗前后CT对照结果见图1。持续口服阿法替尼靶向治疗1年，期间患者出现轻度腹泻、黏膜炎、皮疹、口腔炎等不良反应。患者于2024年7月复查出现肿瘤进展（45.6 mm×32.0 mm），建议使用抗*NTRK*融合的靶向治疗方案。但沟通后患者和家属放弃了靶向治疗，选择了放疗和化疗，目前患者已接受1个周期放疗[大体肿瘤靶体积（gross tumor volume, GTV）原发灶，GTV为GTV外扩及周围受累淋巴结，处方剂量：95%PTV 60 Gy/2 Gy/30 F，95%FGTV 72 Gy/2.4 Gy/30 F]和2个周期化疗（培美曲塞二钠700 mg联合卡铂600 mg *ivgtt q21d*），治疗相对顺利。

## 2 讨论

近年来NSCLC的治疗取得了极大的进展，尤其是靶向治疗的应用，明显提高了患者治疗的客观缓解率，延长了无进展生存时间，并显著提高了患者的生活质量。分子分型是NSCLC实施靶向治疗的基础。*EGFR* G719X为罕见的NSCLC的*EGFR*突变类型（定位于*EGFR*基因的外显子18号）。G719X突变在NSCLC所有*EGFR*突变中的发生率为1.53%。*EGFR* G719A点突变导致719位的甘氨酸被丙氨酸取代，从而导致EGFR的组成性活化^[5]^。研究^[7⇓-9]^表明*EGFR* G719A突变对吉非替尼（Gefitinib）、厄洛替尼（Erlotinib）和奥希替尼（Osimertinib）的反应低于*EGFR*外显子19缺失突变的细胞，但对第二代EGFR-TKIs敏感。因此，建议第二代EGFR-TKIs作为G719X突变晚期NSCLC患者的首选。

*NTRK*融合是多种成人和儿童肿瘤的致癌驱动因素，有研究^[3]^表明*NTRK*基因融合在NSCLC中出现概率为0.1%-3.3%，表1^[10⇓⇓⇓⇓⇓⇓⇓⇓-19]^总结了各项研究中*NTRK*基因融合在NSCLC中的变异概率。*NTRK*融合变异通常作为第一代和第三代EGFR-TKIs的潜在耐药机制出现。一项对3050例*EGFR*突变阳性NSCLC样本的调查^[20]^中，通过配对治疗前后样本证实了*NTRK*融合变异在第一代EGFR-TKIs（厄洛替尼）治疗后出现。另外，在第三代EGFR-TKIs耐药的肺癌患者中也检测到了*NTRK*融合^[21,22]^。NSCLC中的原发性*NTRK*融合变异模式仍不是很清楚，但似乎与其他驱动因子突变和融合互斥。一项研究^[10]^调查了11例*NRTK*融合的NSCLC患者的临床及病理特征发现，所有患者缺乏典型驱动突变，虽然其中6例被识别为共突变，但没有发现与*EGFR*、间变性淋巴瘤激酶（anaplastic lymphoma kinase, *ALK*）或c-ros原癌基因1-受体酪氨酸激酶（c-ros oncogene 1-receptor tyrosine kinase, *ROS1*）等常见致癌基因的共突变。本案例是原发性*EGFR* G719A突变和*LMNA-NTRK1*融合共同存在的肺腺癌病例的首次报道。*LMNA-NTRK1*融合变异可使LMNA的卷曲二聚结构域与NTRK1的酪氨酸激酶结构域融合，导致下游信号通路的异常激活，进而导致肿瘤的发生^[23]^。

**表1:** NTRK基因融合在NSCLC中的变异概率

Reference	Tissue-type	Total variation rate of NTRK (%)	NTRK gene	Case number	Fusion variation ratio (%)
Farago AF, et al^[10]^	NSCLC	0.23 (11/4872)	NTRK1	6	0.12
			NTRK2	1	0.02
			NTRK3	4	0.08
Solomon JP, et al^[11]^	LUAD	0.23 (9/3993)	/	/	/
Westphalen CB, et al^[12]^	NSCLC	0.24 (136/56,615)	/	/	/
Gatalica Z, et al^[13]^	LUAD	0.1 (4/4073)	NTRK1	1	0.02
			NTRK2	1	0.02
			NTRK3	2	0.05
Rosen EY, et al^[14]^	LUAD	0.16 (6/3658)	/	/	/
Si X, et al^[15]^	NSCLC	0.59 (44/7395)	/	/	/
Forsythe A, et al^[16]^	NSCLC	0.17 (-/-)	/	/	/
Vaishnavi A, et al^[17]^	LC	3.3 (3/91)	/	/	/
Seker-Cin H, et al^[18]^	NSCLC	0.36 (2/5554)	NTRK1	1	0.02
			NTRK2	1	0.02
de Oliveira Cavagna R, et al^[19]^	NSCLC	1.36 (2/147)	NTRK1	1	0.68
			NTRK3	1	0.68

NSCLC: non-small cell lung cancer; LUAD: lung adenocarcinoma; LC: lung cancer.

大多数*NTRK*融合阳性的NSCLC患者在诊断时有转移^[3]^。TRK抑制剂如拉罗替尼（Lorlatinib）和恩曲替尼（Entrectinib）对于具有*NTRK*融合的局部晚期或转移性肿瘤患者显示出显著的功效和良好的安全性，已被美国食品药品监督管理局（Food and Drug Administration, FDA）批准用于治疗*NTRK*融合阳性实体瘤。EGFR-TKIs与TRK抑制剂联合应用可能是*NTRK*融合介导的EGFR-TKIs耐药患者的可选治疗之一^[3]^。病例研究^[4]^显示，*EGFR*（19del）突变型NSCLC患者EGFR-TKIs耐药后出现获得性*LMNA-NTRK1*融合变异，使用*NTRK*融合抑制剂恩曲替尼（Entrectinib）联合EGFR-TKIs奥希替尼治疗后5个月，尽管胰腺转移缓慢进展但腹膜转移有所缓解。广泛的文献调研^[4]^发现，EGFR-TKIs耐药后同时阻断*EGFR*突变和耐药后获得性*NTRK*融合可为临床提供益处，然而，联合疗法的有效性和安全性需要进一步研究。

由于EGFR在皮肤生理学中的关键作用，皮肤不良反应在使用EGFR-TKIs的患者中很常见。研究^[24]^表明，接受靶向EGFR-TKIs治疗的NSCLC患者药物识别能力低下，与严重皮肤不良反应发生率高有关。此外，合并症、营养状态、血液白细胞介素-6水平及联合应用药物等因素也与皮肤不良反应的严重程度有关^[24]^。本例患者使用阿法替尼后出现皮疹等不良反应，可能与药物识别能力有关。

本文首次报道了1例罕见的原发性*EGFR *G719A突变合并*LMNA-NTRK1*融合变异的NSCLC，并初步探讨了该基因融合可能为EGFR-TKIs的潜在耐药机制。然而，*NTRK* 融合与其他典型突变互斥，临床结果数据较少，个体治疗仍有待通过累积病例和进一步的研究来证实。使用NGS来鉴定患者的突变模式对于*EGFR*突变耐药的NSCLC的个性化治疗和管理至关重要。

## References

[1] Hagopian G, Nagasaka M. Oncogenic fusions: targeting *NTRK*. Crit Rev Oncol Hematol, 2024, 194: 104234. doi: 10.1016/j.critrevonc.2023.104234 38122917

[2] Frampton JE. Entrectinib: a review in *NTRK*+ solid tumours and *ROS1*+ NSCLC. Drugs, 2021, 81(6): 697-708. doi: 10.1007/s40265-021-01503-3 33871816 PMC8149347

[3] Liu F, Wei Y, Zhang H, et al. *NTRK* fusion in non-small cell lung cancer: diagnosis, therapy, and TRK inhibitor resistance. Front Oncol, 2022, 12: 864666. doi: 10.3389/fonc.2022.864666 35372074 PMC8968138

[4] Wang JL, Wang LS, Zhu JQ, et al. Survival benefit of combinatorial osimertinib rechallenge and entrectinib in an *EGFR*-mutant NSCLC patient with acquired *LMNA-NTRK1* fusion following osimertinib resistance. Respirol Case Rep, 2022, 10(11): e01054. doi: 10.1002/rcr2.1054 36258694 PMC9574602

[5] Sehgal K, Rangachari D, VanderLaan PA, et al. Clinical benefit of tyrosine kinase inhibitors in advanced lung cancer with *EGFR*-G719A and other uncommon *EGFR* mutations. Oncologist, 2021, 26(4): 281-287. doi: 10.1002/onco.13537 32969527 PMC8018319

[6] Xia H, Xue X, Ding H, et al. Evidence of *NTRK1* fusion as resistance mechanism to EGFR TKI in *EGFR*+ NSCLC: results from a large-scale survey of *NTRK1* fusions in Chinese patients with lung cancer. Clin Lung Cancer, 2020, 21(3): 247-254. doi: 10.1016/j.cllc.2019.09.004 31761448

[7] Kobayashi Y, Togashi Y, Yatabe Y, et al. *EGFR* exon 18 mutations in lung cancer: molecular predictors of augmented sensitivity to Afatinib or Neratinib as compared with first- or third-generation TKIs. Clin Cancer Res, 2015, 21(23): 5305-5313. doi: 10.1158/1078-0432.Ccr-15-1046 26206867

[8] Tu HY, Ke EE, Yang JJ, et al. A comprehensive review of uncommon *EGFR* mutations in patients with non-small cell lung cancer. Lung Cancer, 2017, 114: 96-102. doi: 10.1016/j.lungcan.2017.11.005 29173773

[9] Yang JC, Sequist LV, Geater SL, et al. Clinical activity of afatinib in patients with advanced non-small-cell lung cancer harbouring uncommon *EGFR* mutations: a combined post-hoc analysis of LUX-Lung 2, LUX-Lung 3, and LUX-Lung 6. Lancet Oncol, 2015, 16(7): 830-838. doi: 10.1016/s1470-2045(15)00026-1 26051236

[10] Farago AF, Taylor MS, Doebele RC, et al. Clinicopathologic features of non-small-cell lung cancer harboring an *NTRK* gene fusion. JCO Precis Oncol, 2018, 2: PO. 18.00037. doi: 10.1200/po.18.00037 PMC6132056 30215037

[11] Solomon JP, Linkov I, Rosado A, et al. *NTRK* fusion detection across multiple assays and 33,997 cases: diagnostic implications and pitfalls. Mod Pathol, 2020, 33(1): 38-46. doi: 10.1038/s41379-019-0324-7 31375766 PMC7437403

[12] Westphalen CB, Krebs MG, Le Tourneau C, et al. Genomic context of *NTRK1/2/3* fusion-positive tumours from a large real-world population. NPJ Precis Oncol, 2021, 5(1): 69. doi: 10.1038/s41698-021-00206-y 34285332 PMC8292342

[13] Gatalica Z, Xiu J, Swensen J, et al. Molecular characterization of cancers with *NTRK* gene fusions. Mod Pathol, 2019, 32(1): 147-153. doi: 10.1038/s41379-018-0118-3 30171197

[14] Rosen EY, Goldman DA, Hechtman JF, et al. *TRK* fusions are enriched in cancers with uncommon histologies and the absence of canonical driver mutations. Clin Cancer Res, 2020, 26(7): 1624-1632. doi: 10.1158/1078-0432.CCR-19-3165 31871300 PMC7124988

[15] Si X, Pan R, Ma S, et al. Genomic characteristics of driver genes in Chinese patients with non-small cell lung cancer. Thorac Cancer, 2021, 12(3): 357-363. doi: 10.1111/1759-7714.13757 33300283 PMC7862783

[16] Forsythe A, Zhang W, Phillip Strauss U, et al. A systematic review and *meta*-analysis of neurotrophic tyrosine receptor kinase gene fusion frequencies in solid tumors. Ther Adv Med Oncol, 2020, 12: 1758835920975613. doi: 10.1177/1758835920975613 PMC7758559 33425024

[17] Vaishnavi A, Capelletti M, Le AT, et al. Oncogenic and drug-sensitive *NTRK1*rearrangements in lung cancer. Nat Med, 2013, 19(11): 1469-1472. doi: 10.1038/nm.3352 24162815 PMC3823836

[18] Seker-Cin H, Tay TKY, Kazdal D, et al. Analysis of rare fusions in NSCLC: genomic architecture and clinical implications. Lung Cancer, 2023, 184: 107317. doi: 10.1016/j.lungcan.2023.107317 37586177

[19] de Oliveira Cavagna R, de Andrade ES, Tadin Reis M, et al. Detection of *NTRK* fusions by RNA-based nCounter is a feasible diagnostic methodology in a real-world scenario for non-small cell lung cancer assessment. Sci Rep, 2023, 13(1): 21168. doi: 10.1038/s41598-023-48613-4 38036758 PMC10689426

[20] Schrock AB, Zhu VW, Hsieh WS, et al. Receptor tyrosine kinase fusions and BRAF kinase fusions are rare but actionable resistance mechanisms to EGFR tyrosine kinase inhibitors. J Thorac Oncol, 2018, 13(9): 1312-1323. doi: 10.1016/j.jtho.2018.05.027 29883838

[21] Piotrowska Z, Isozaki H, Lennerz JK, et al. Landscape of acquired resistance to Osimertinib in *EGFR*-mutant NSCLC and clinical validation of combined EGFR and RET inhibition with Osimertinib and BLU-667 for acquired *RET* fusion. Cancer Discov, 2018, 8(12): 1529-1539. doi: 10.1158/2159-8290.Cd-18-1022 30257958 PMC6279502

[22] Helman E, Nguyen M, Karlovich CA, et al. Cell-free DNA next-generation sequencing prediction of response and resistance to third-generation EGFR inhibitor. Clin Lung Cancer, 2018, 19(6): 518-530.e7. doi: 10.1016/j.cllc.2018.07.008 30279111

[23] Bender J, Anderson B, Bloom DA, et al. Refractory and metastatic infantile fibrosarcoma harboring *LMNA-NTRK1* fusion shows complete and durable response to crizotinib. Cold Spring Harb Mol Case Stud, 2019, 5(1): a003376. doi: 10.1101/mcs.a003376 30709876 PMC6371745

[24] Du R, Yang H, Zhou H, et al. The relationship between medication literacy and skin adverse reactions in non-small-cell lung cancer patients undergoing targeted EGFR-TKI therapy. BMC Cancer, 2022, 22(1): 491. doi: 10.1186/s12885-022-09599-w 35505288 PMC9066960

